# An internal expectation guides *Drosophila* egg-laying decisions

**DOI:** 10.1126/sciadv.abn3852

**Published:** 2022-10-28

**Authors:** Vikram Vijayan, Zikun Wang, Vikram Chandra, Arun Chakravorty, Rufei Li, Stephanie L. Sarbanes, Hessameddin Akhlaghpour, Gaby Maimon

**Affiliations:** Laboratory of Integrative Brain Function and Howard Hughes Medical Institute, The Rockefeller University, New York, NY, USA.

## Abstract

To better understand how animals make ethologically relevant decisions, we studied egg-laying substrate choice in *Drosophila*. We found that flies dynamically increase or decrease their egg-laying rates while exploring substrates so as to target eggs to the best, recently visited option. Visiting the best option typically yielded inhibition of egg laying on other substrates for many minutes. Our data support a model in which flies compare the current substrate’s value with an internally constructed expectation on the value of available options to regulate the likelihood of laying an egg. We show that dopamine neuron activity is critical for learning and/or expressing this expectation, similar to its role in certain tasks in vertebrates. Integrating sensory experiences over minutes to generate an estimate of the quality of available options allows flies to use a dynamic reference point for judging the current substrate and might be a general way in which decisions are made.

## Teaser

*Drosophila* integrate sensory experiences over minutes to form an estimate of the quality of substrate options for egg laying.

## INTRODUCTION

When trying to identify the best fruit in a basket, each inspected item updates our internal expectation of what the basket has to offer. We use this expectation to settle for an unripe option, for example, if none of the items inspected over the past few seconds to minutes have been ripe. The process of updating one’s expectations based on recent experiences is central to many daily decisions. Might we be able to study such an expectation process in a tractable model system, like *Drosophila* (*1*–*3*)?

Female *Drosophila* lay dozens of eggs, one at a time, over a few hours. During the past decades, researchers have studied *Drosophila*’s egg-laying preferences across a broad array of conditions (*4*–*15*). In foundational work on sucrose substrates, it was shown that flies do not simply express innate preferences for specific sucrose concentrations. Instead, flies treat the same sucrose substrate as attractive or repulsive depending on the nature of the other option in a simple two-choice chamber (*8*, *9*). This observation suggests that flies can assess the relative value of two sucrose substrates in deciding where to lay eggs. To better understand this relative valuation process, we performed a detailed analysis of the egg-laying behavior of *Drosophila* in simple two-choice chambers as well as in chambers with more than two substrate options or where they experienced options for very different lengths of time. The results argue that *Drosophila* form an internal expectation about their environment—akin to the memory of previously inspected fruits in the human example—which guides their egg-laying decisions. The impact of this expectation on behavior is evident, typically, for few minutes after flies encounter a new substrate. We argue via behavioral genetic experiments that dopamine neurons and D1-like dopamine receptors participate in the expectation process.

## RESULTS

### Flies can make a relative value decision between two sucrose-containing substrates

**Fig. 1.**
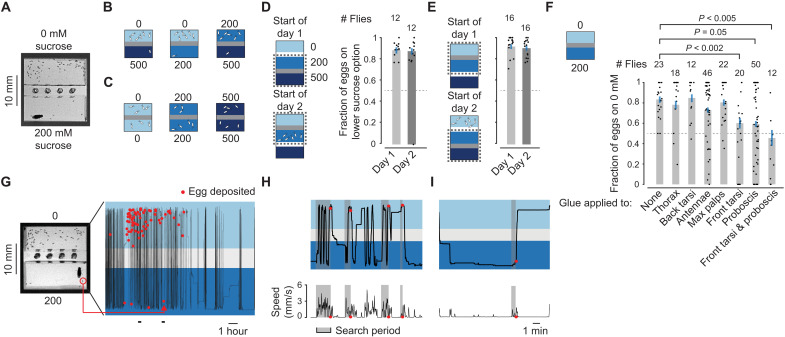
Flies continuously assess the relative sucrose concentration of substrates, using their tarsi and proboscis, to guide egg-laying decisions. (**A**) Egg-laying choice chamber with fly. (**B** and **C**) Schematic of relative value choice data from (*16*). Each drawn egg represents approximately six eggs in a real experiment. Numbers indicate sucrose concentration in mM. (**D** and **E**) Fraction of eggs on the lower sucrose option with 95% confidence interval. Each dot represents one fly. Dotted line at 0.5 is drawn for reference (Materials and Methods). In (D) and (E), flies laid an average of 40, 42, 42, and 44 eggs per fly. (**F**) Fraction of eggs on the lower sucrose option with 95% confidence interval. Each dot represents one fly. Flies laid an average of 25, 24, 29, 25, 29, 18, 23, and 14 eggs per fly. *P* values are calculated using the two-sided Wilcoxon rank sum test. *P* values are *P* < 0.005, *P* < 0.005, and *P* < 0.001 (top to bottom), calculated using a subset of the displayed data (flies that laid ≥5 eggs). Using flies that laid ≥5 eggs prevents flies that laid just a few eggs from biasing the distribution of fractions in either direction. Eighteen, 13, 12, 41, 20, 13, 28, and 9 flies laid ≥5 eggs. All *P* values for flies that laid ≥5 eggs reach a significance level of *P* < 0.05 after a Bonferroni correction for seven comparisons (*P* < 0.05/7 = 0.007). (**G**) *Y* position trajectory and egg deposition events from a single fly in a high-throughput egg-laying choice chamber. (**H**) Zoomed-in view of the first time period indicated at the bottom of the time series in (G). (**I**) Zoomed-in view of the second, later time period. Locomotor speed in (H) and (I) is smoothed with a 5-s boxcar filter. Traces in (G) to (I) are from data collected in (*16*).

We placed single wild-type Canton-S (CS) flies, overnight, in dark, custom egg-laying substrate choice chambers where agarose-based substrate islands are separated by a 2.5-mm-wide plastic barrier (Fig. 1A and fig. S1A) (*16*). The 2.5-mm barrier is approximately the length of the fly, which means that the fly’s legs touched either one substrate or the other, but rarely (if ever) touched both substrates at the same time. In all experiments, agarose substrates contained 1.6% ethanol and 0.8% acetic acid, simulating a rotting fruit (*4*). Substrates additionally contained varying amounts of sucrose.

We focused on substrates that varied in their sucrose concentrations (*8*, *9*) for two reasons. First, because flies cannot see or smell sucrose at a distance, if flies changed their egg-laying rate on one sucrose substrate due to the existence of a second, we could be sure that this was due to a historical effect of experiencing the second substrate and not due to them currently sensing the distant substrate. Second, previous work has extensively demonstrated that flies make a relative comparison between two sucrose-containing substrates (*8*, *9*, *16*). That is, if flies are only allowed to lay eggs on 0, 200, or 500 mM sucrose substrates, all are acceptable; however, when flies are allowed to choose between any pair of such options, they strongly prefer the lower concentration (schematized in Fig. 1, B and C) (*16*). Thus, for example, the same 200 mM option is avoided when paired with 0 mM (schematized in Fig. 1B, middle) and preferred when paired with 500 mM (schematized in Fig. 1B, right).

Previous work has demonstrated that the preference for lower sucrose is not due to flies entirely avoiding the higher sucrose option (*9*, *16*). The preference is also not explained by a competing drive, like feeding, preventing egg laying on higher sucrose, because flies spend a similar amount of time on high and low sucrose options before laying an egg and we do not observe them to be extending their proboscis (to eat) more when walking over the higher sucrose option before egg laying (*16*). Spatial memory of the location of substrate options does not seem to serve a prominent role in guiding egg laying in these chambers either (*16*). What does seem to explain much of the variance in the flies’ egg-laying choices is the time history of how different sucrose concentrations are experienced, as we describe below.

A preference for lower, rather than higher, sucrose may seem counterintuitive because fly larvae (which emerge from the eggs) can metabolize sucrose for energy. However, a soft substrate with relatively low sucrose and high ethanol simulates a rotting fruit (*7*), which is the preferred egg-laying environment of most drosophilids (*4*). Specifically, the rotting portions of a fruit are relatively depleted of sucrose compared to the ripe parts—due to fermentation of sugars into alcohol—and these regions could be the ideal place for *Drosophila melanogaster* to lay eggs.

### Flies continuously make sucrose assessments of their environment to guide egg-laying decisions

Sucrose-sensing gustatory receptors have been implicated in egg-laying choice (*7*–*9*, *17*), suggesting that our flies continuously assessed the local sucrose concentration to guide behavior. However, an alternative possibility is that flies assessed the sucrose concentrations of substrates only at the start of an hours-long experiment and then relied on their initially laid eggs or other chemicals, like deposited pheromones, to guide subsequent egg-laying decisions. Male flies have been shown to deposit pheromones that guide female egg laying (*18*), making it plausible that females might do likewise. To test whether substrate marking serves a role in our female-only experiments, we measured egg-laying choice in chambers where we converted a preferred substrate to a nonpreferred one, and vice versa, by changing the nature of a second option (fig. S1B). For example, we let individual flies lay eggs overnight in 200 mM versus 500 mM chambers (Fig. 1D, left bar). The next day, we removed the flies, closed off the 500 mM islands, and opened access to 0 mM islands. We kept the chambers at 4°C to prevent eggs from hatching and then equilibrated the chambers to 24°C before assaying another set of flies overnight. Flies laid eggs normally on 0 mM, although 200 mM had many eggs from the start (Fig. 1D, right bar). Thus, our flies did not seem to rely on previously laid eggs, or other long-lasting chemical cues, which mark either the preferred (Fig. 1D) or nonpreferred (Fig. 1E) option, to guide substrate choice.

Which sensory organs do flies use for assessing the sucrose content of substrates to guide egg laying? We covered various parts of the fly’s body with light-curable glue (*19*, *20*), likely rendering these parts unable to sense the external chemical environment (*21*). We then assessed egg laying in 0 mM versus 200 mM chambers. When we applied glue to the front tarsi (front leg tips) or proboscis, the bias to laying eggs on 0 mM was significantly reduced, and when both these body parts had glue, we could not detect a population-level preference for 0 over 200 mM (Fig. 1F). Choice was not significantly affected, however, when we applied glue to the back tarsi (rear leg tips), for example, indicating that applying glue, in general, does not affect the ability of flies to target the low sucrose option for egg laying. Overall, these data argue that flies continuously assess the sucrose content of substrates via receptors on their front tarsi and proboscis to guide egg-laying decisions.

### Two hypotheses

How do flies develop their bias toward laying eggs on the lower sucrose option? One possibility is that flies decrease their egg-laying probability each time they sense an increase in sucrose, and vice versa. Another option is that the flies remember the properties of substrate options previously encountered in a format that allows for quantitative comparison with the sucrose concentration of the current substrate. Such a working memory of past substrates—which we will call an expectation—might allow flies to adjust their egg-laying probability based on how the sucrose concentration of the current substrate compares to the expectation. We reasoned that a careful analysis of the spatiotemporal dynamics of egg-laying behavior might allow us to differentiate between these hypotheses.

### A framework for understanding how previous substrate experiences guide egg-laying choices

We tracked the *xy* position and egg deposition events of flies in our chambers (*16*) and plotted the *y* position over time (Fig. 1, G to I, and table S1). A fly was deemed to reside on one of the two substrates based on whether its centroid was above or below the chamber’s midline. Past work that tracked flies during egg laying (*9*, *22*) used chambers in which flies could walk on the hard walls and ceiling (where flies do not lay eggs), and only the *xy* position of the centroid was analyzed, making the flies’ precise substrate experiences ambiguous. Because flies could not walk on the ceiling or side walls of our chambers (*16*), when their centroid was over a substrate, they were physically standing on that substrate.

Before laying an egg, flies increase their locomotion during a so-called search period (*8*, *16*). The search period begins after ovulation (*16*), i.e., after an egg is passed from an ovary to the uterus. It is during the search that flies seem to be actively determining on which substrate to lay an egg. To quantitatively define the search epoch preceding each egg, we used a simple algorithm to find the time window before each egg-laying event in which the fly’s locomotor activity was elevated (Fig. 1, H and I, gray sections) (Materials and Methods) (*16*).

To examine how a fly’s substrate history affects egg laying, we calculated the fly’s egg-laying rate, during the search period, as a function of time since a substrate transition (*16*). We focused on the egg-laying rate during the search period so that periods of time without an ovulated egg would not affect our rate values (e.g., when the fly might be sleeping in our very long experiments). That said, all our conclusions are robust to varying definitions of the search period (fig. S2). Analyzing hundreds or thousands of eggs was important for accurately estimating rate functions, and thus, we typically combined data from all tested flies in the same chamber type to generate these curves. We believe that this simplification is reasonable because all combined flies had the same genetic background and flies with outlier behavior were not obvious in visual inspection of the data.

**Fig. 2.**
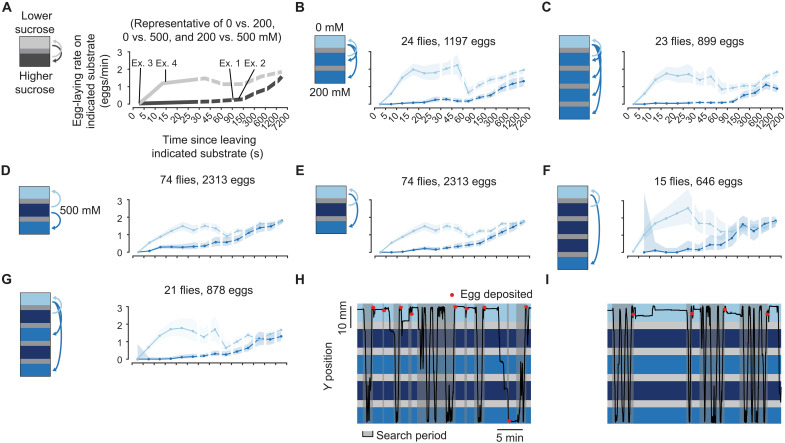
Flies lay most of their eggs on the best of three substrate options. (**A**) Schematic of the mean egg-laying rate during the search period as a function of time following a substrate transition (time zero). The rate on each of the two substrates is shown separately (see main text and Materials and Methods). This schematic is based on data from (*16*) and is representative of 0 mM versus 200 mM, 0 mM versus 500 mM, and 200 mM versus 500 mM chambers. The light gray curve represents the egg-laying rate on the lower sucrose option as a function of time since last visiting the higher sucrose option (i.e., the transition represented by the light gray arrow on the schematic). Conversely, the dark gray curve represents the egg-laying rate on the higher sucrose option as a function of time since last visiting the lower sucrose option. (**B** to **G**) Mean egg-laying rate curves during the search period on a substrate as a function of time since visiting another substrate. Ninety percent confidence intervals are calculated using the Clopper-Pearson method (Materials and Methods) and are shaded. In (F) and (G), confidence intervals for 200 mM are large (or missing) for short times because flies rarely (or never) quickly transit between 0 and 200 mM because of the distance between the substrates. (**H** and **I**) Example traces from two separate flies.

To aid interpretation of the egg-laying rate functions, let us consider some schematic curves from a previous study (*16*) (Fig. 2A). A hypothetical fly finishes ovulating and initiates a search on high sucrose 90 s after last experiencing low sucrose. The average egg-laying rate is ~0.2 eggs/min (Fig. 2A, Ex. 1). The fly searches on high sucrose for 30 s, and the egg-laying rate is still low, ~0.2 eggs/min (Fig. 2A, Ex. 2). The fly then transitions to low sucrose (Fig. 2A, Ex. 3). After 15 s, the egg-laying rate is six times higher, ~1.2 eggs/min (Fig. 2A, Ex. 4), and this hypothetical fly deposits an egg. Note that in this analysis, flies make use of substrate experiences regardless of whether these experiences occurred during the current search or not. This interpretation is consistent with the observation that in two-choice sucrose chambers, flies that start a search on higher sucrose know, somehow, to leave that substrate (flies leave higher sucrose in 964 of 1205 or 80% of searches initiated on higher sucrose), whereas flies that start a search on lower sucrose know to stay (flies leave lower sucrose in only 918 of 2744 or 33% of searches initiated on lower sucrose) (*P* < 0.001, two-sided Wilcoxon rank sum test; Materials and Methods) (*16*). In addition, note that our egg-laying rate plots do not consider how long a fly has been searching. This simplification seems reasonable because flies still lay eggs on low sucrose even after very long searches that have many transitions (fig. S3). Last, note that all rate functions, even ones associated with the low-sucrose substrate, start off with a low rate for the first 10 s after a transition. This initially low rate is observed, at least in part, because flies do not lay eggs on the plastic barrier between substrates (*16*) and because they are, by definition, walking and not pausing to lay an egg during a transition (*16*).

Our previously reported result based on these egg-laying rate functions is that when flies transition to high sucrose in two-choice chambers, the egg-laying rate is near 0 for the first ~2 min (Fig. 2A, darker curve) and then slowly rises to approach the rate on low sucrose (Fig. 2A, darker and lighter curves merge) (*16*). The fact that the egg-laying rates on high and low sucrose become similar over time explains why flies lay eggs similarly in chambers containing only one sucrose option, even when that option is high in sucrose (Fig. 1C) (*16*).

### Flies lay eggs on the best of three options

As mentioned earlier, in two-choice chambers, flies could, in theory, simply assess whether the sucrose concentration has increased or decreased after each transition. Alternatively, flies might use an internal expectation about substrates. To test between these two hypotheses, we increased the number of substrate options. First, we designed three- and five-island chambers (fig. S1, C and D) and confirmed that in these larger chambers, flies effectively chose the low sucrose option when presented with two sucrose concentrations, and they did so with similar egg-laying rate dynamics to those observed in standard two-choice chambers (Fig. 2, B and C). We then constructed a three-choice chamber with a 500 mM substrate in the middle and 0 and 200 mM substrate on either side (Fig. 2D). In this chamber, a fly that lays eggs after any decrease in sucrose concentration should lay eggs upon entering either 0 or 200 mM from 500 mM. However, we observed that flies inhibited egg laying upon entering 200 mM in this chamber (Fig. 2D and table S1). As time passed since the last visit to 0 mM, the egg-laying rate on 200 mM gradually increased (Fig. 2E). (The darker blue line in Fig. 2E is the rate on 200 mM since visiting 0 mM, whereas in Fig. 2D it is the rate on 200 mM since visiting 500 mM.) These results were robust to increasing the physical distance between the 0 and 200 mM options (Fig. 2F) as well as to chambers in which flies experienced several, locally attractive, increases in acceptability (i.e., decreases in sucrose concentration from 500 to 200 mM) along their path (Fig. 2, G to I, and movie S1). In all these three-choice chambers, flies inhibited egg laying nearly completely on 200 mM if they had visited 0 mM in the last ~30 s (Fig. 2, E to G). This strong inhibition is remarkable because it means that flies were treating 200 mM as a very poor option in these three-choice chambers, although 200 mM is strongly preferred when flies made the identical substrate transition (from 500 to 200 mM) in two-choice chambers (Fig. 2A) (*16*). That said, the 0 and 200 mM egg-laying rate functions did appear to merge at an earlier time point in three-choice (Fig. 2, E to G) compared to two-choice chambers (Fig. 2, A to C), suggesting that some aspect of the three-choice chamber makes differentiating substrates for extended periods more challenging for flies.

### Flies lay eggs more rapidly on high-quality substrates that they visit rarely or briefly

If flies can keep track of the option with highest relative value in multichoice chambers, might they also be able to keep track of how likely they are to experience a good option in a given environment? For example, if a fly rarely experiences a 0 mM substrate, would it lay an egg more quickly when it does experience it?

**Fig. 3.**
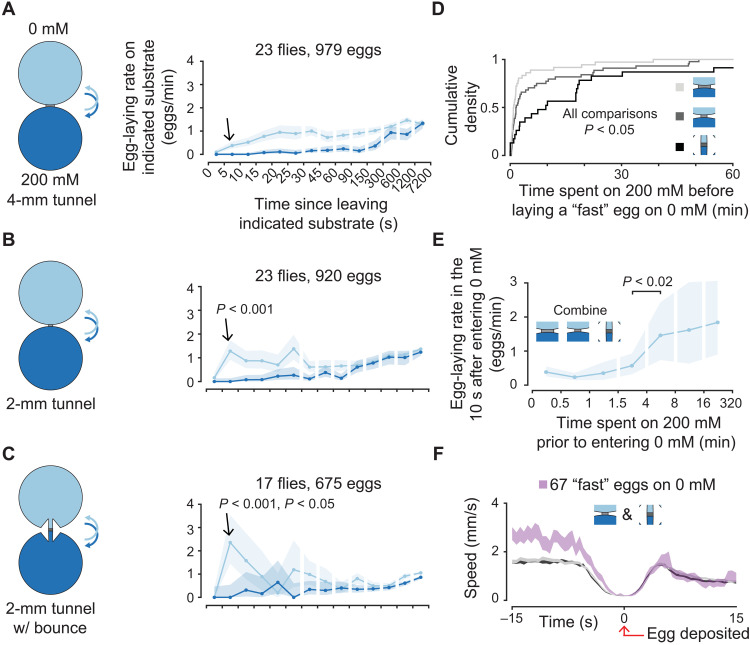
Flies lay eggs faster upon entering a preferred substrate that they have not experienced recently. (**A** to **C**) Mean egg-laying rate during the search period on a substrate as a function of time since visiting another substrate. The 90% confidence interval is shaded. *P* values are calculated using the two-sided Wilcoxon rank sum test (Materials and Methods). *P* value in (B) is comparing the indicated bin to the same bin in (A). *P* values in (C) are comparing the indicated bin to the same bin in (A) and (B). (**D**) Cumulative distribution of time spent on 200 mM before entering 0 mM and laying an egg in 10 s or less (i.e., “fast” eggs). Medians are 54, 96, and 576 s for increasing shades of gray. Thirty-six, 44, and 23 eggs for increasing shades of gray. *P* values are calculated using the two-sided Wilcoxon rank sum test. (**E**) Mean egg-laying rate during the search period in the 10 s after entering 0 mM as a function of time previously spent on 200 mM. The 90% confidence interval is shaded. (**F**) Mean locomotor speed aligned to egg deposition. Data in gray are all eggs from the chambers, as colored in (D). For increasing shades of gray, we analyzed the following number of eggs: 979, 918 [two less than (B) because eggs that were laid in the first or last few minutes of a video were not included in all triggered averages to prevent averaging of partial traces], and 675.

We designed chambers where flies would experience 200 mM for a long time before entering 0 mM (fig. S1, E to G). In chambers with a tiny (2 mm) tunnel between substrates, flies laid eggs at a higher rate in the 5- to 10-s window after a transition to 0 mM compared to very similar chambers with a 4-mm tunnel (Fig. 3, A and B, bin indicated with an arrow). To further restrict flies from entering the 2-mm tunnel, we designed a chamber with angled walls near the tunnel to “bounce” flies that are walking along the edge of the chamber away from the tunnel. In this chamber, flies laid eggs even more rapidly upon entering the 0 mM side (Fig. 3C, bin indicated with an arrow, and movie S2).

Do flies lay eggs quickly on the 0 mM substrate because they have experienced the relatively worse 200 mM substrate for a long time? As expected, in the chambers shown in Fig. 3 (A to C), flies tended to spend progressively more time on the 200 mM substrate before laying “fast” eggs (eggs laid within 10 s of entering 0 mM) (Fig. 3D). Similarly, when we combine all the egg-laying events from these three chambers, we observe that flies increase their egg-laying rate upon entering 0 mM specifically if they have not experienced 0 mM for >~4 min (Fig. 3E). The time delay between the dip in the flies’ locomotor speed and egg deposition is similar for fast eggs (Fig. 3F, pink) and all eggs in these three chambers (Fig. 3F, shades of gray), suggesting that the decision to lay an egg, and not the egg deposition motor program, is made faster. Note that the absolute locomotor speed before egg laying is higher for fast eggs because flies need to be moving to cross the barrier between substrates. These results suggest that flies make faster egg-laying decisions when they find an unexpected, preferred substrate.

**Fig. 4.**
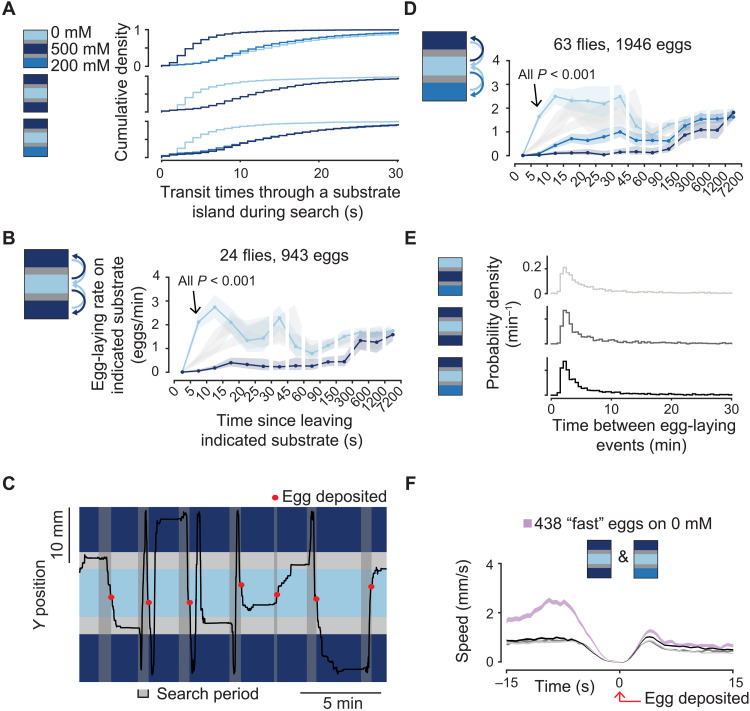
Flies lay eggs faster upon entering a preferred substrate that they typically visit briefly. (**A**) Cumulative distribution of transit times through substrate islands during the search period. In total, 2637, 5694, and 2903 transit times for 0, 500, and 200 mM, respectively, in the 0/500/200 chamber. In total, 1625 and 1508 transit times for 0 and 500 mM, respectively, in the 500/0/500 chamber. In total, 1741, 3354, and 1810 transit times for 500, 0, and 200 mM, respectively, in the 500/0/200 chamber. (**B**) Mean egg-laying rate during the search period on a substrate as a function of time since visiting another substrate. The 90% confidence interval is shaded. The 90% confidence interval of 0 mM egg-laying rate curves from 0/200/200 (Fig. 2B), 0/500/200 (Fig. 2D), and 0/500/500 chambers is shaded in gray for reference. *P* value is calculated using the two-sided Wilcoxon rank sum test (Materials and Methods) and is comparing the indicated bin to the same bins in light gray traces. *P* value is also <0.001 if compared to a 0/500/500 mM chamber (table S1). (**C**) Example trace. (**D**) Mean egg-laying rate as in (B). (**E**) Time between egg-laying events. Medians are 5.1, 4.1, and 4.4 min. In total, 2262, 922, and 1901 inter-egg intervals, respectively. (**F**) Mean locomotor speed aligned to egg deposition. Data in gray are all eggs from the chambers as colored in (E). In total, 2312, 943, and 1946 eggs for increasing shades of gray.

We noticed that flies often circled our chambers while staying adjacent to the walls (thigmotaxis), which meant that they had shorter transit times through the middle islands of rectangular chambers (median, ~4.5 s) compared to the edge islands (median, ~13 s) (Fig. 4A). We thus posited that if the preferred substrate were in the briefly visited middle island, flies may need to make a faster decision to lay an egg on that substrate. We found that flies lay eggs more quickly after a transition to the preferred substrate in these chambers (Fig. 4B, bin indicated with an arrow) compared to three-island chambers where the preferred substrate was on either edge (Fig. 4B, gray confidence intervals). This result was clear in individual trajectories (Fig. 4C and movie S3). Concomitant with faster egg laying on the middle substrate, flies also had a higher egg-laying rate than expected (from our other three-choice experiments) on the closest alternative (compare medium blue curve from 0 to 90 s in Fig. 4D with Fig. 2E). Such anomalous eggs on an intermediate option may be undesirable, and minimizing them may be one of the reasons why flies resort to quick egg laying only when necessary (i.e., when a good substrate is experienced only briefly) (see Discussion).

The inter-egg interval is not increased when the preferred substrate is in the middle island, suggesting that flies are not abandoning egg-laying search attempts in these chambers (which would artificially increase egg-laying rates if these abandoned search attempts are not included in our search period definition) (Fig. 4E). Furthermore, the increased egg-laying rate with briefly visited substrates holds for broader definitions of the search period (fig. S2E). As in our previous experiments, the egg deposition motor program (as proxied by the dip in locomotor speed) is not clearly accelerated (Fig. 4F). Flies thus decide to lay eggs faster on the best option in environments where they typically visit that option only briefly.

Together, these data support the hypothesis that flies form an expectation regarding the substrate options in their environment rather than simply responding to proximal changes in the sucrose concentration. The flies’ expectation seems to keep track of the best option experienced over the past few minutes (Fig. 2) alongside of how rarely or briefly a substrate is experienced (Figs. 3 and 4). Models that attempt to explain the observed behavior as a function of sucrose concentration alone, without a comparison to an internal expectation, fail to reproduce important aspects of the data (fig. S4 and Materials and Methods).

### Different *Drosophila* strains and species show different egg-laying rates in the same environment

**Fig. 5.**
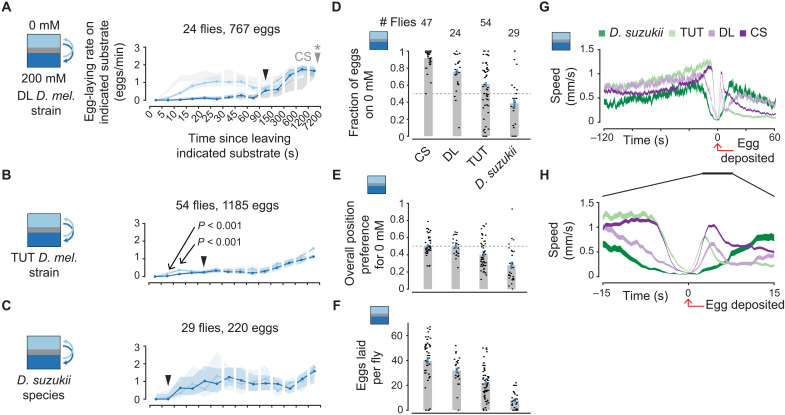
Different *Drosophila* strains and species show different egg-laying rates in the same environment. (**A** to **C**) Mean egg-laying rate during the search period on a substrate as a function of time since visiting another substrate. The 90% confidence interval is shaded. (A) Ninety percent confidence intervals of 0 and 200 mM egg-laying rate curves for CS flies in a 0 mM versus 200 mM chamber in light and medium gray, respectively, for comparison [reproduced from (*16*)]. *P* values are calculated using the two-sided Wilcoxon rank sum test (Materials and Methods) and are comparing 0 and 200 mM for the indicated bin. Filled arrows indicate first bin past 5 s for which *P* > 0.05 (i.e., a quantitative estimate of when the curves merge). Filled arrow for CS flies is marked with a star because the 0 and 200 mM egg-laying rate curves do not merge within the plotted interval. (**D**) Fraction of eggs on 0 mM with 95% confidence interval. Each dot represents one fly. (**E**) Fraction of time spent on 0 mM per fly. Each dot represents one fly. (**F**) Eggs laid per fly. Each dot represents one fly. (**G** and **H**) Mean locomotor speed aligned to egg deposition. In total, 214, 1184, 766, and 1862 eggs for *D. suzukii*, TUT, DL, and CS, respectively. In (D) to (H), CS data are from (*16*).

How flies interpret substrate experiences for egg laying may differ depending on the needs and preferences of specific strains or species. We tested two other wild-type strains of *D. melanogaster* in 0 mM versus 200 mM chambers and found that both released inhibition of egg laying on 200 mM faster than did CS flies (Fig. 5, A and B, filled arrows indicate the time when 0 and 200 mM egg-laying rate curves merge). For example, the TUT strain showed no statistically detectable evidence of substrate history after only ~20 s of being on a new substrate (Fig. 5B). One interpretation is that each wild-type strain readjusts its expectations on a time scale that is tuned to the statistics of the niche from which it was isolated. For example, flies that tend to experience frequent fluctuations in substrate quality in their natural environment may readjust their expectations more slowly—i.e., hold out longer for a better option—than those that do not. Alternatively, certain fly strains may be more capable than others at building or using expectations due to genetic constraints whose nature is not yet clear.

Most species of *Drosophila* prefer to lay eggs on rotten fruit (*4*). However, *Drosophila suzukii* prefer to lay eggs on ripe fruit (*4*, *23*). A variety of evolutionary changes have enabled this switch in *D. suzukii*, including an enlarged and serrated ovipositor for penetrating ripe fruit (*23*). We found that, unlike the three wild-type strains of *D. melanogaster*, *D. suzukii* did not exhibit a measurable egg-laying preference for lower sucrose substrates in our paradigm, i.e., the egg-laying rates on 0 and 200 mM were similar in a two-choice chamber (Fig. 5C). *D. suzukii* actually laid slightly more eggs on the higher (200 mM) sucrose substrate (Fig. 5D), consistent with their overall preference toward spending more time on high sucrose (Fig. 5E). A loss of preference for low sucrose may be an adaptation that helps *D. suzukii* lay eggs on ripe fruit. In general, different strains/species laid a different number of eggs (Fig. 5F), which may be indicative of their egg-laying tendencies in the wild or their sensitivity to our experimental conditions. All strains/species, on average, increase their locomotor speed (search) before pausing to deposit an egg (Fig. 5, G and H). However, qualitative differences in locomotor speed exist between each strain/species, and they exhibit different time delays between the dip in average locomotor speed and egg deposition (*P* < 0.001 all comparisons, two-sided Wilcoxon rank sum test; Fig. 5H and Materials and Methods). It is likely that, for example, *D. suzukii* has a longer egg deposition motor program aimed to penetrate harder, ripe fruit. Overall, these results suggest that aspects of the expectation processes may be evolving with the needs and preferences of different *Drosophila*.

### Flies do not strongly modulate their egg-laying rates in all substrate choice environments

Inspired by recent work demonstrating that *D. melanogaster* prefer to lay eggs on softer substrates (*4*, *24*, *25*), we sought to identify whether CS flies may strongly decrease their egg-laying rate for several minutes when they encounter a harder substrate option, much like they do when they encounter an option with higher sucrose concentration (Fig. 2A) (*16*). Because substrate stiffness, like sucrose concentration, cannot be sensed at a distance, it is amenable to our substrate history–dependent egg-laying rate analysis.

**Fig. 6.**
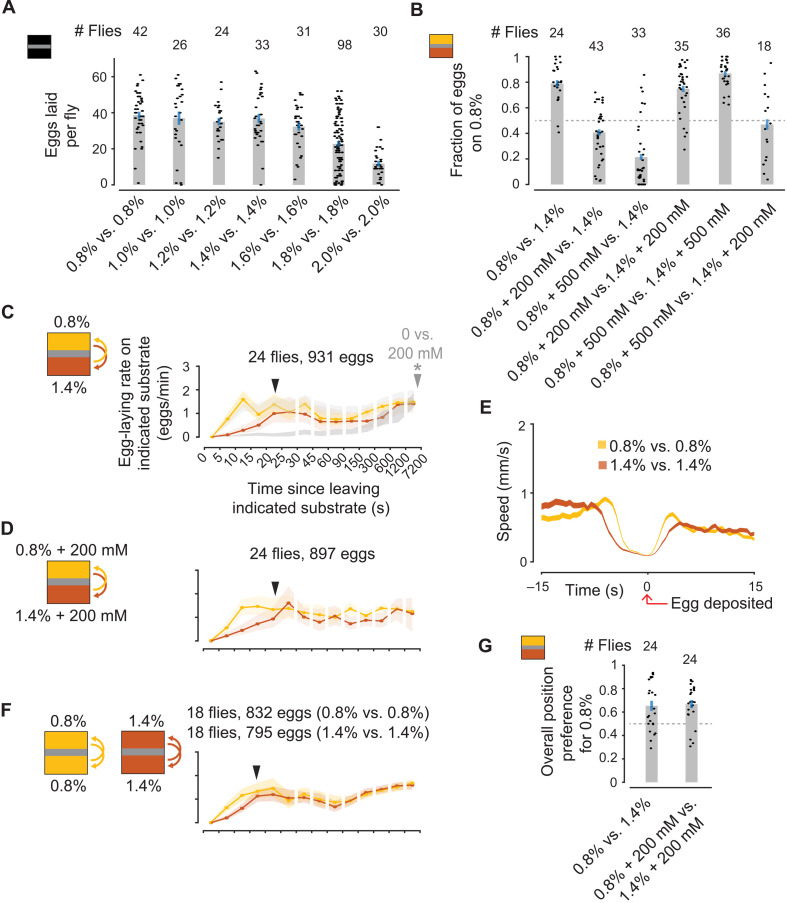
Flies do not appreciably modulate egg-laying rates in all substrate choice environments. (**A**) Eggs laid per fly. Each dot represents one fly. (**B**) Fraction of eggs on 0 mM with 95% confidence interval. Each dot represents one fly. Flies laid an average of 38, 36, 38, 36, 30, and 33 eggs per fly. (**C** and **D**) Mean egg-laying rate during the search period on a substrate as a function of time since visiting another substrate. The 90% confidence interval is shaded. (C) Ninety percent confidence intervals of 0 and 200 mM egg-laying rate curves for CS flies in a 0 mM versus 200 mM chamber in light and medium gray, respectively, for comparison [reproduced from (*16*)]. *P* values are calculated using the two-sided Wilcoxon rank sum test (Materials and Methods) and are comparing 0.8 and 1.4% for the indicated bin. Filled arrows indicate first bin past 5 s for which *P* > 0.05 (i.e., a quantitative estimate of when the curves merge). Filled arrow for 0 mM versus 200 mM is marked with a star because the 0 and 200 mM egg-laying rate curves do not merge within the plotted interval. (**E**) Mean locomotor speed aligned to egg deposition. In total, 831 and 795 eggs for 0.8% versus 0.8% and 1.4% versus 1.4%, respectively. (**F**) Mean egg-laying rate as in (C) and (D), but for chambers with the same option on both sides. (**G**) Fraction of time spent on 0.8% per fly. Each dot represents one fly.

We assayed egg laying on substrates with different percentages of agarose (Fig. 6A). Substrates made with a higher percentage of agarose are stiffer than those with a lower percentage (*26*). We paired two distinct agarose percentages upon which flies readily lay eggs—0.8 and 1.4%—and found that, as expected, flies prefer to lay eggs on the softer option (Fig. 6B, first bar). Adding sucrose to the softer option shifts the preference to the harder option (Fig. 6B, bars 2 and 3), and adding equal amounts of sucrose to both options has little effect (Fig. 6B, bars 4 and 5). Adding 500 mM sucrose to the softer option and 200 mM sucrose to the harder option yields ~50% of eggs on both sides, on average (Fig. 6B, bar 6). These experiments suggest that the difference between 0.8 and 1.4% agarose is roughly comparable, at the level of fraction of eggs laid on the preferred option, to the sucrose concentrations we used in our previous experiments. However, different from sucrose choice, flies did not strongly repress egg laying for several minutes upon entering the harder option (Fig. 6C). The egg-laying rates on 0.8 and 1.4% agarose merge within just ~20 s of being on a new substrate (Fig. 6C, filled arrow), and this result is robust to adding equal amounts of sucrose to both options (Fig. 6D, filled arrow).

We noticed that flies pause for a longer duration to lay eggs on harder substrates (*P* < 0.001, two-sided Wilcoxon rank sum test; Fig. 6E and Materials and Methods). Because a longer pause indicates that it might take longer to deposit an egg after a transition onto a hard substrate, we assessed the egg-laying rate after a transition across the plastic boundary in two-choice chambers loaded with the same substrate on both sides. We find that egg-laying rates are lower for ~15 s after crossing the boundary in a 1.4% versus 1.4% chamber compared to a 0.8% versus 0.8% chamber. This indicates that at least some of the repression of egg laying in ~20 s after transitioning from 0.8 to 1.4% (Fig 6, C and D) is likely to reflect the biomechanical constraints of laying eggs in 1.4% agarose rather than substrate memory. [Note that flies pause similarly when laying eggs on 1% agarose substrates with 0, 200, and 500 mM sucrose (*16*), indicating that, as expected, sucrose and hardness were treated differently by *Drosophila*—i.e., increasing sucrose was not simply increasing hardness.] If flies only gently and transiently repress egg laying on the harder substrate in 0.8% versus 1.4% chambers, how is it that they lay ~80% of their eggs on the softer option (Fig. 6B)? At least part of the answer is that flies simply spend more time on the softer option, which is expected to increase the fraction of eggs laid on that option independent of any expectation-related adjustment of egg-laying rates (Fig. 6G). Overall, these results demonstrate that not all substrate modalities lead to similar expectation-dependent changes in egg-laying rates for a given *Drosophila* strain, suggesting that either not all modalities are incorporated into expectation-related calculations or not all expectations similarly modulate egg-laying rates.

### Dopa decarboxylase–expressing neurons are a strong candidate for performing expectation-related calculations

**Fig. 7.**
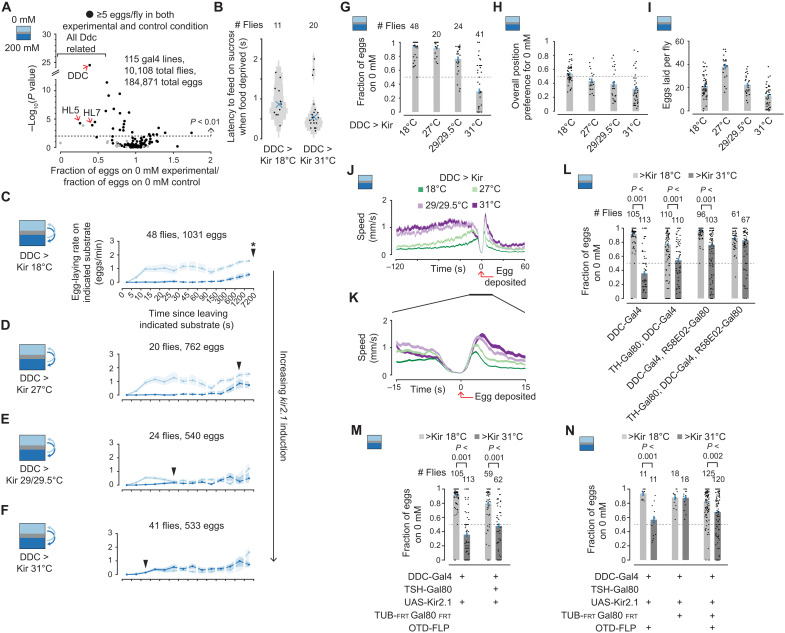
Dopamine neurons are a strong candidate for performing expectation-related calculations. (**A**) Scatterplot of individual Gal4 lines assayed. The *x* axis represents the fraction of eggs laid on 0 mM, after pooling all eggs from a given experimental genotype, divided by the same value for control flies. The *y* axis shows the *P* value calculated using the two-sided Wilcoxon rank sum test for the difference between the experimental and control groups. The top three Gal4 lines with ≥5 eggs per fly in both the experimental and control condition are labeled. (**B**) Latency to feeding by food-deprived flies after crossing the plastic barrier from 0 to 200 mM (Materials and Methods). Each dot represents one fly. Median is indicated with an x. (**C** to **F**) Mean egg-laying rate during the search period on a substrate as a function of time since visiting another substrate. The 90% confidence interval is shaded. Filled arrows are an estimate of when the curves merge as in Fig. 5A. (**G**) Fraction of eggs on 0 mM with 95% confidence interval. Each dot represents one fly. (**H**) Fraction of time spent on 0 mM per fly. Each dot represents one fly. (**I**) Eggs laid per fly. Each dot represents one fly. (**J** and **K**) Mean locomotor speed aligned to egg deposition. In total, 1029, 761, 540, and 533 eggs for 18°, 27°, 29°/29.5°, and 31°C, respectively. (**L** to **N**) Fraction of eggs on 0 mM as in (H). Flies laid an average of 19, 7, 19, 11, 28, 16, 27, 21, 19, 7, 17, 11, 25, 30, 21, 22, 21, and 23 eggs per fly. *P* values are calculated using the two-sided Wilcoxon rank sum test, and all displayed *P* values are <0.001 using a subset of the displayed data (flies that laid ≥5 eggs). In (A) to (N), all flies have tubulin promoter driving Gal80^ts^.

To identify neurons that might be involved in the expectation process, we screened for neurons whose activity contributes to normal 0 mM versus 200 mM sucrose egg-laying choice. We reduced the excitability of genetically targeted neurons by expressing in them, via the Gal4/UAS system, the Kir2.1 potassium channel (*27*). A temperature-sensitive Gal80 transgene (*28*) was used to restrict expression of *kir2.1* until a day before the assay. Control and experimental flies were siblings with the identical genotype, but experimental flies were held at 31°C, instead of 18°C for controls, for a 23-hour period before the egg-laying assay, during which *kir2.1* gene expression was permitted. Egg-laying assays for all genotypes, experimental and controls, were performed at the same, 24°C, temperature (Materials and Methods). The Gal4 lines tested in this screen were chosen either (i) randomly, (ii) based on previous egg-laying studies (*8*–*10*, *29*), or (iii) based on hits identified earlier in the screen. Of the 115 Gal4 lines tested, in the control condition, 110 (96%) laid more eggs on 0 than 200 mM (fig. S5A) and 111 (97%) laid five or more eggs per fly (fig. S5B). To visualize the results of the screen, we plotted the *P* value versus fold change in choice (experimental choice divided by control choice) (Fig. 7A). Of the 115 Gal4 lines tested, the top 8 hits, and many of the hits just outside of the top 8, had the Gal4 transgene driven by a 3,4-dihydroxyphenylalanine (Dopa) decarboxylase (Ddc)–related enhancer (table S2). We performed more detailed experiments on the DDC-Gal4 line (*30*), specifically, to see whether we could better understand how these neurons contribute to egg-laying choice.

First, as a control, we checked whether DDC-Gal4 experimental flies—hereafter, DDC > Kir (31°C pre)—could still sense sucrose. We assayed proboscis extension of food-deprived flies in 0 mM versus 200 mM chambers with a movable barrier between substrates (fig. S1H and Materials and Methods). Flies that were not food-deprived, regardless of pretreatment, showed no measurable proboscis extension upon entering 200 mM. DDC > Kir (31°C pre) food-deprived flies either extended their proboscis or, in cases where our camera could not see their proboscis clearly, entered an obvious feeding posture quickly after crossing onto 200 mM (Fig. 7B and fig. S6). Thus, DDC > Kir (31°C pre) flies can still sense the sucrose content of a substrate, suggesting that the role of Ddc neurons is likely downstream of the sucrose receptors.

We induced *kir2.1* at four different levels in the DDC-Gal4 neurons, using four temperatures of pretreatment to remove varying amounts of the Gal80^ts^ inhibition (Fig. 7, C to F). The more Kir2.1 we expressed, the earlier in time after a substrate transition did the 0 and 200 mM egg-laying rate curves begin to merge (Fig. 7, C to F, filled black arrows). When Kir2.1 expression was strongest in DDC > Kir (31°C pre) flies, the egg-laying rate curves were nearly superimposable throughout (Fig. 7F), meaning that these flies laid eggs similarly on 0 and 200 mM substrates. The DDC > Kir phenotype is different from one where flies shift preference to sucrose or generally increase variability in behavior because, in these two interpretations, the 200 mM egg-laying rate curve should increase equally in all bins regardless of the time since a transition. Instead, flies show properly biased egg-laying rates proximal to a substrate transition in the two intermediate pretreatment temperatures (27° and 29°/29.5°C) (Fig. 7, D and E). We interpret these results to mean that expectation-related information—either the value of the expectation or the comparison of this signal with the value of the current substrate (i.e., a relative value signal)—is degraded when Ddc neurons are made leakier with Kir2.1. The overall egg-laying rate during search tended to drop as Kir2.1 expression was increased (progressively lower mean curve levels in Fig. 7, D to F), meaning that flies searched for longer before laying an egg. This overall drop in the egg-laying rate is also consistent with the notion that relative value signals are compromised with Kir2.1 expression. As expected from the egg-laying rate curves, we found that increased induction of *kir2.1* resulted in a progressive decrease in the fraction of eggs laid on 0 mM (Fig. 7G). The fact that DDC > Kir (31°C pre) flies laid more eggs on 200 mM than on 0 mM is consistent with them spending more time, overall, on the 200 mM side (Fig. 7H) alongside their indifference as to where to lay eggs (Fig. 7F). The decrease in egg-laying preference of DDC > Kir flies (Fig. 7, C to F) was unrelated to the number of eggs laid (Fig. 7I) or to changes in the egg deposition motor program (as proxied by the dip in locomotor speed) (Fig. 7, J to K).

### Dopamine neurons within the Ddc-expressing population may be important

The Ddc enzyme is involved in the biosynthesis of both dopamine and serotonin. In vertebrates, certain populations of dopaminergic neurons are involved in expectation-related calculations (*31*–*36*). To test whether the dopamine neurons within the DDC-Gal4 population were important for choice, we used TH-Gal80 (tyrosine hydroxylase–related enhancer) (*37*) and/or R58E02-Gal80 (dopamine transporter–related enhancer) (*38*) transgenes to minimize or eliminate Kir2.1 expression in the dopaminergic subset of cells targeted in the DDC-Gal4 line. Expression of Gal80 by either of the abovementioned enhancers partially rescued the choice defect in DDC > Kir (31°C pre) flies. With both enhancers driving Gal80, the choice defect was fully rescued (Fig. 7L). These results are consistent with the hypothesis that the normal electrical activity of dopamine neurons, within the set of cells targeted by the DDC-Gal4 line, is relevant for choice. The fact that TH-Gal80 or R58E02-Gal80 alone does not rescue the choice defect completely suggests that many dopamine neurons may be involved. However, it is also formally possible that each Gal80 transgene progressively decreases Kir2.1 expression in the critical, small subset of DDC-Gal4 neurons.

Neurons in which the enhancer for Ddc is active are found in the *Drosophila* brain and ventral nerve cord (fig. S7). To assess whether neurons in the brain are important for choice, we used two genetic methods to bias Kir2.1 expression to DDC-Gal4–positive neurons in the brain (Fig. 7, M and N). Flies still retain an egg-laying choice defect after Kir2.1 expression in the ventral nerve cord is minimized with TSH-Gal80 (Fig. 7M) (*39*) or when expression is biased to the central brain using OTD-FLP in conjunction with TUB-FRT Gal80 FRT (Fig. 7N) (*40*). This indicates that DDC-Gal4–positive neurons in the brain are important for egg-laying choice. However, in both cases, the magnitude of the choice defect appears reduced (compare Fig. 7M, bar sets 1 to 2, and Fig. 7N, bar sets 1 to 3), suggesting that neurons in the ventral nerve cord may serve a role among other possibilities like that the genetic manipulations yielded incomplete or off-target effects or that the (necessarily) different genetic backgrounds of the experimental flies in these experiments are associated with quantitatively altered sucrose preferences. (We normally keep genetic backgrounds matched in comparisons to avoid such concerns.)

### Dopamine signaling via Dop1R1 and Dop1R2 are important for expectation-related calculations

**Fig. 8.**
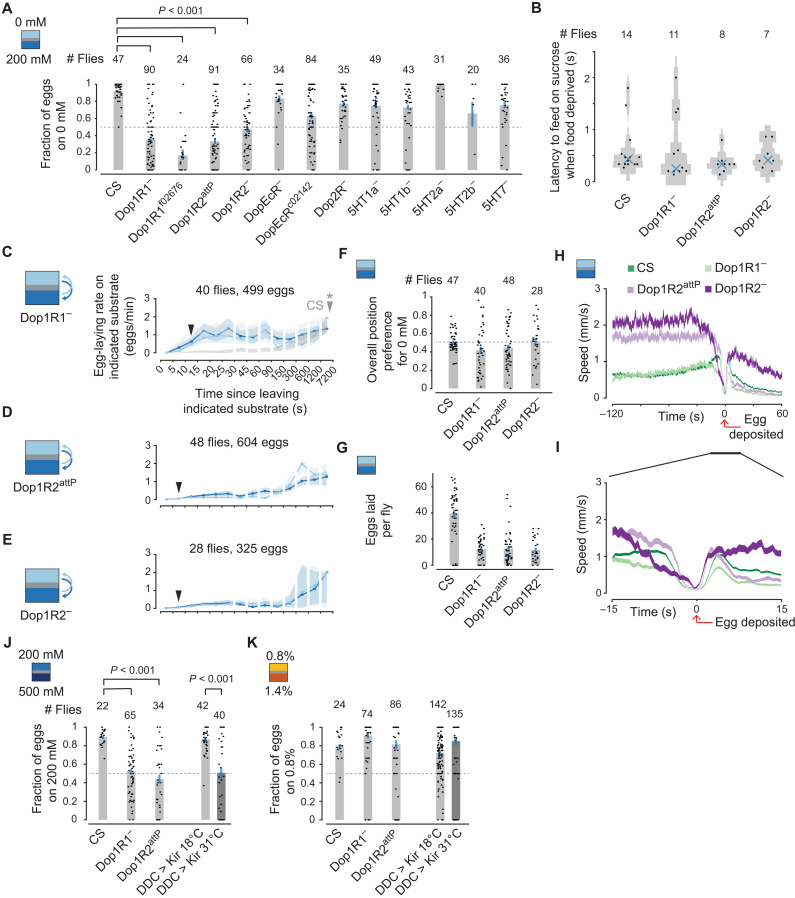
Dopamine signaling via Dop1R1 and Dop1R2 is important for expectation-related calculations. (**A**) Fraction of eggs on 0 mM with 95% confidence interval. Each dot represents one fly. Flies laid an average of 40, 14, 7, 10, 10, 11, 12, 23, 15, 25, 19, 2, and 16 eggs per fly. 5HT2b^−^ flies lay very few eggs, on average. *P* values are calculated using the two-sided Wilcoxon rank sum test, and all displayed *P* values are <0.001 using a subset of the displayed data (flies that laid ≥5 eggs). (**B**) Latency to feeding by food deprived as in Fig. 7B. (**C** to **E**) Mean egg-laying rate during the search period on a substrate as a function of time since visiting another substrate. The 90% confidence interval is shaded. (C) Ninety percent confidence intervals of 0 and 200 mM egg-laying rate curves for CS flies in gray as in Fig. 5A. Filled arrows are an estimate of when the curves merge as in Fig. 5A. (**F**) Fraction of time spent on 0 mM per fly. Each dot represents one fly. (**G**) Eggs laid per fly. Each dot represents one fly. (**H** and **I**) Mean locomotor speed aligned to egg deposition. In total, 1862, 498, 586, and 323 eggs for CS, Dop1R1^−^, Dop1R2^attP^, and Dop1R2^−^, respectively. (**J** and **K**) Fraction of eggs on 200 mM (J) or 0.8% (K) with 95% confidence interval. Each dot represents one fly. Flies laid an average of 45, 15, 12, 17, 6, 38, 6, 3, 11, and 2 eggs per fly, respectively. *P* values as in (A). These genotypes lay fewer eggs, overnight, in chambers without a food source (e.g., sucrose) but still lay a large fraction of their eggs on 0.8%. DDC > Kir flies have tubulin promoter driving Gal80^ts^. In (A) and (F) to (I), CS data are from (*16*).

To further test the role of dopamine, we assayed mutants for all four dopamine receptors (*41*) and all five serotonin (5HT) receptors (*42*) in 0 mM versus 200 mM egg-laying choice (Fig. 8A). We used putative null mutants that were generated by deleting or replacing specific exons—Dop1R1^−^ (*43*), Dop1R2^attP^ (*44*), Dop1R2^−^ (*41*), DopEcR^−^ (*41*), Dop2R^−^ (*41*), 5HT1a^−^ (*42*), 5HT1b^−^ (*42*), 5HT2a^−^ (*42*), 5HT2b^−^ (*42*), 5HT7^−^ (*42*) as well as strong hypomorphs generated via random transposon insertion—DopEcR^c02142^ (*45*–*47*) and Dop1R1^f02676^ (*47*–*49*). All mutants were homozygous and in a CS background (Materials and Methods). Both mutants of either of the two *Drosophila* dopamine D1–like receptors, Dop1R1 and Dop1R2 (*50*), decreased the fraction of eggs laid on 0 mM below 0.5 similar to the effect observed in DDC > Kir (31°C pre) flies (Fig. 7G, last bar). Mutants of other receptors did not bring choice below 0.5.

We performed more detailed experiments on all three of the putative null mutants for either Dop1R—Dop1R1^−^, Dop1R2^attP^, and Dop1R2^−^. When starved, all three mutant genotypes initiate feeding after touching sucrose with their front tarsi, indicating that they retain the ability to generally sense sucrose (Fig. 8B). Dop1R1 mutant flies showed similar egg-laying rate curves on 0 and 200 mM, highlighting an inability to compare the two options (Fig. 8C). Likewise, Dop1R2 mutant flies showed similar rate curves on 0 and 200 mM sucrose (Fig. 8, D and E). However, the egg-laying rate curves for Dop1R1 and Dop1R2 mutants appear qualitatively different. Both the 0 and 200 mM rate curves for Dop1R1 mutants look more like the 0 mM rate curve for CS flies (Fig. 8C, light gray shaded curve), while both the 0 and 200 mM rate curves for Dop1R2 mutants look more like the 200 mM rate curve for CS flies (Fig. 8C, dark gray shaded curve). This difference might hint at different roles for these receptors in expectation-related calculations. The fact that Dop1R1^−^ and Dop1R2^attP^ flies laid more eggs on 200 mM than on 0 mM is consistent with them spending more time, overall, on the 200 mM side (Fig. 8F) alongside their roughly similar egg-laying rates for both substrates (Fig. 8, C and D). All three mutants, on average, lay a similar number of eggs (Fig. 8G) and increase their locomotor speed (search) before pausing to deposit an egg (Fig. 8, H and I).

Dop1R1 mutant, Dop1R2 mutant, and DDC > Kir (31°C pre) flies are, as expected, also defective in 200 mM versus 500 mM sucrose choice (Fig. 8J). The defect may appear to be smaller in magnitude than that in 0 mM versus 200 mM sucrose (compare to Fig. 8A and Fig. 7G); however, we posit that this is because the mutants and DDC > Kir (31°C pre) flies no longer have a positional bias to the higher sucrose side when both options have some sucrose, leading to a 50-50 distribution of eggs when egg-laying rates on both options are similar. Furthermore, consistent with a role for dopamine in expectation-dependent modulation of egg-laying rates, and not egg laying in general, these flies are not defective in 0.8% versus 1.4% agarose choice (Fig. 8K).

## DISCUSSION

We found that *Drosophila* egg-laying decisions are affected by past substrate experiences and that they can be understood via a framework in which flies internally construct an expectation of the substrate composition of their environment. This internal expectation is then compared to the quality of the current substrate to guide egg-laying decisions.

### A model for expectation-guided egg laying

**Fig. 9.**
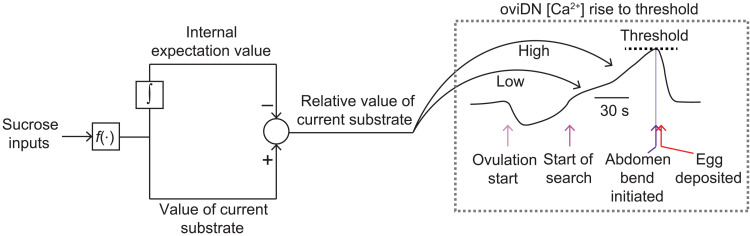
A model for expectation-guided egg laying. The integral symbol indicates the brain integrating the value of sucrose sensory inputs over time to build an internal expectation signal. The “*f*()” block represents the process by which the brain converts [sucrose] to value. The internal expectation value signal is compared to the value of the current substrate to create a relative value signal. The comparison is diagrammed as a subtraction, but other comparison operations are possible. The relative value signal regulates a rise to threshold process in the oviDNs to drive egg laying.

A set of descending neurons, called oviDNs (oviposition descending neurons), is required for laying eggs (*51*). We have recently imaged calcium signals in oviDNs, and their activity dips during ovulation, fluctuates up and down over many seconds to minutes during search, and crosses a threshold level immediately before the final abdomen bend for egg laying (*16*). On average, the rate of rise of the oviDN calcium signal during search is modulated by the relative value of the current substrate. We hypothesize that an internal expectation signal—inferred to exist from the experiments discussed here—is compared to an estimate of the value of the current substrate, with the difference between these two variables playing a role in the oviDN signal’s propensity to rise and thus its likelihood to hit threshold (Fig. 9). For example, when a fly encounters an unexpected, preferred substrate, the difference in value between the current substrate and the expectation is large and, as a result, the slope of the oviDN calcium rise is large. A large slope causes the oviDNs to hit threshold quickly, resulting in a fast decision to lay an egg. Whereas current physiological experiments (*16*) have not yielded sufficient data with long periods on high sucrose before transitioning onto plain, future work at the neurophysiological level should test for such effects.

### An internal sense of the external environment that guides behavior

An internally constructed expectation that allows an animal to change its response to the same stimulus based on an informed prediction is a signature of cognitive behavior (*52*). One way to conceptualize our findings is that flies latently learn (i.e., learn without obvious positive or negative reinforcement) (*53*, *54*) an internal representation of their external environment, which shapes responses to future stimuli. Several studies in *Drosophila* have shown that adult flies and larvae use sensory experiences over the past few seconds, which were not obviously reinforced, to regulate behavior, including during courtship (*55*), taxis (*56*), and spatial orientation (*57*). Similar short time scale comparisons have been observed even in *Escherichia coli* during chemotaxis (*58*, *59*). The longer, minute time scale behavioral changes we observe here suggest that historical information may be converted into an explicit, stable internal expectation signal in the fly brain, which future neurophysiological work should aim to reveal.

What form might an internal expectation take? One possibility is that flies build an explicit mental model of their environment based on a remembered list of the substrates they previously visited, augmented with the times these substrates were encountered and the durations spent on each. A perhaps more realistic possibility is that flies convert their substrate experiences into an analog neural signal, or a set of such signals, which encode an expectation value of the environment. For example, a single neuron could track this expectation explicitly via its spike rate, built off recent substrate experiences. In a 0 mM versus 500 mM versus 200 mM chamber (Fig. 2E), this expectation neuron’s spike rate would be continuously high (assuming the fly has recently visited 0 mM). The egg-laying rate would thus be low unless the fly is situated on 0 mM, which is the only substrate that would drive other neurons that track the value of the current substrate, to spike at a high enough rate to exceed the high rate of the expectation neuron (Fig. 9). If the same fly were transferred to a 500 mM versus 0 mM versus 200 mM chamber (Fig. 4D), the expectation neuron’s spike rate would drop because the best option is experienced more briefly. This lower spike rate would make the current-minus-expectation output high enough on both 0 and 200 mM to cause an increase in the egg-laying probability on both options, explaining the faster egg laying on 0 mM and the increased, anomalous eggs on the closest alternative. Note that this putative expectation neuron’s spike rate may need to track more than just the mean sucrose concentration experienced over the past few minutes because if flies were merely using such a mean signal they would, for example, have had a hard time inhibiting egg laying on the 200 mM option in 0-500-500-500-200 mM chambers (Fig. 2F) (also see fig. S4 and Materials and Methods). Although a single expectation neuron is possible, it is important to note that our experiments point to a wider array of neurons being involved in the expectation process (Fig. 7L).

Does the internal expectation process that we infer here incorporate sensory modalities other than sucrose sensing? We imagine that the answer is yes, because flies likely evolved to use all relevant information to make the best egg-laying decisions possible (*60*). However, additional work will be needed to test this idea. Specifically, other modalities might be combined with sucrose to form a single, unified expectation signal or independent expectation signals for different modalities might exist, for example. Our work with 0.8% versus 1.4% agarose (Fig. 6) argues that an appreciable expectation-dependent change in egg-laying rates need not be observed for all substrate modalities, even in cases where flies lay more eggs on one option over another. These observations highlight that the sucrose results are nontrivial. Our egg-laying rate analysis framework, which was needed for revealing an internal expectation process, is not limited to studying egg laying in flies and could be applied to choice behavior in other animals. The sucrose concentrations we used—200 and 500 mM—are in the range of sugar content found in fruits, like bananas (*61*), suggesting that our observations on sucrose-related expectations are not an artifact of using unusually high concentrations, raising the prospects that expectations with other naturally occurring modalities will be discovered, down the road. Our experiments with substrate hardness were confined to a particular substrate combination (0.8% versus 1.4%) and a specific strain (CS), and future work with other combinations of agarose percentages, strains, and species may discover that *Drosophila* can build and use expectations of substrate hardness to modulate egg-laying rates. That said, our data are also consistent with the possibility that flies have an absolute stiffness set point—at ~2.0% agarose—above which they do not lay many eggs. It is possible that with stiffness, larvae simply cannot survive above a certain level––perhaps because they cannot burrow or eat effectively––whereas with different sucrose concentrations all options support basic survival but some options are quantitatively better than others. With an absolute set point for a variable, as may be the case with stiffness, there would be no need to perform a relative comparison of options. In a situation where a dynamic set point is useful, as with sucrose, a relative value comparison would make more functional sense to implement. Future work will be needed to test this hypothesis.

### Dopamine neurons and expectations

Monoaminergic neurons have been implicated in increasing or decreasing the fraction of eggs laid on a preferred substrate (*9*, *10*, *29*). However, this past work did not provide a computational framework with which to interpret the role of these neurons in egg-laying decisions. A clear hypothesis for the computations involved in egg-laying substrate choice (Fig. 9) will be invaluable for interpreting neurophysiological signals (*62*), especially if the relevant signals turn out to be distributed across a broad set of neurons, as our data suggest (Fig. 7L).

A recent report has argued that Parkinson’s disease patients have impairments in their ability to use priors (i.e., expectations) for decision-making (*63*), which is reminiscent of the behavioral phenotype we observe when Ddc-expressing neurons are inhibited in egg-laying *Drosophila*. Parkinsonian patients suffer from a progressive loss of midbrain dopamine neurons. In vertebrates, at least some dopamine neurons signal a reward prediction error (*31*–*33*), which represents the difference between the value of a current experience and the expectation of value associated with that experience. It is believed that these reward prediction error signals act both to update expectations about the world and to modify ongoing behavior (*34*–*36*). A reward prediction error is akin to the proposed relative value output signal in our model (Fig. 9). If dopamine neurons carried such a signal in egg-laying flies—and thus modified the fly’s expectations about the world (pathway not diagrammed in Fig. 9) and/or the rate of rise of the oviDN signal to more directly affect ongoing action—it would make sense why their activity is critical for egg-laying substrate choice. Dopamine signals in *Drosophila* have been shown to track the value of certain states in a seemingly absolute manner (*38*, *64*, *65*), and it is possible that future neurophysiological work will reveal that fly dopamine neurons also signal value in a relative manner (*66*). The apparently opposing roles of Dop1R1 and Dop1R2 in relative valuation during egg laying (Fig. 8, C to E) means that future work could profitably search for different circuits and molecular pathways that subserve positive and negative assessments of value during this naturalistic behavior, akin to the opposing roles of Dop1R1 and Dop1R2 in fly olfactory learning (*43*, *67*).

The mushroom bodies represent an important associative learning center in the fly brain, and dopaminergic neurons provide the positive or negative valence signals that feed into that structure (*68*). Previous work has shown that hydroxyurea-mediated “ablation” of the mushroom bodies does not affect egg-laying choice (*9*), which suggests, at first pass, that the dopamine neurons involved in egg-laying substrate choice may not function through the mushroom bodies. While we do not know the specific subset of dopamine neurons that are important, genetics-based approaches to minimize Kir2.1 expression in the ventral nerve cord suggest the involvement of neurons in the brain (Fig. 7, M and N) and experiments on proboscis extension suggest that they are likely not affecting sucrose sensory neurons (Figs. 7B and 8B). The pertinent dopamine neurons may reside anywhere in circuitry between taste projection neurons that bring taste information to the brain (*69*)—like TPN2, which has been implicated in sucrose-based egg-laying choice (*17*)—and the oviDNs (*16*, *51*). For example, dopamine neurons could function relatively proximal to the sensory system and control the adaption properties of second-order gustatory neurons (e.g., TPN2) such that they respond when the fly is on the best, recently visited, substrate option.

Although the physiology of dopamine neurons has been well studied in vertebrates (*31*–*36*), many questions remain (*70*). For example, little is known about how expectations are calculated to create reward prediction error signals (*71*–*73*) or how the output of dopamine neurons modulates action (*74*, *75*). Our work suggests that a deeper understanding of the neural basis of egg-laying decisions in the fly brain may help to reveal principles related to value signaling and dopamine physiology, which have proven harder to identify in larger nervous systems.

## MATERIALS AND METHODS

### Flies

Flies were reared on a standard cornmeal medium at 25°C, ambient humidity, and a 12-hour light/12-hour dark cycle. *D. suzukii* were reared with a wet Kimwipe (Kimberly Clark) in the vial or bottle with cornmeal medium. CS flies were obtained from M. Dickinson and were originally from M. Heisenberg. Dickinson Lab (DL) flies were originally from M. Dickinson and were established in 1995 by interbreeding 200 iso-female wild-caught stocks. Tuthill (TUT) flies were originally from J. Tuthill and were established in 2009 by interbreeding 15 wild-caught females (*76*). *D. suzukii* flies were from S. Durkin and L. Zhao and were originally the WT3 strain (*77*). w-; tub Gal80^ts^; UAS Kir2.1 was a gift from N. Yapici. w-; UAS 2xEGFP was backcrossed into the Nippon Project background (for a different study) and was originally from Bloomington Drosophila Stock Center (BDSC) #6874 (*78*). DDC-Gal4 was from BDSC #7009 (*30*). The transgenic genotypes used for the screen, and their sources, are listed in table S2. Dop1R1^−^ (*43*), Dop1R2^attP^ (*44*), DopEcR^−^ (BDSC #84717) (*41*), 5HT1a^−^ (BDSC #86275) (*42*), 5HT1b^−^ (BDSC #86276) (*42*), 5HT2a^−^ (BDSC #86277) (*42*), 5HT2b^−^ (BDSC #86278) (*42*), and 5HT7^−^ (BDSC #86279) (*42*) were backcrossed to a CS background, and DopEcR^c02142^ (*45*, *47*), Dop1R1^f02676^ (*47*, *49*), Dop2R^−^ (BDSC #84716) (*41*), and Dop1R2^−^ (BDSC #84715) (*41*) were backcrossed to a CS background with w^1118^ allele. A single Dop1R1 mutant (BDSC #84714) (*41*) assayed did not have an appreciable choice defect (87% of eggs laid on plain; *n* = 90 flies) and is not included in Fig. 8A for the following reason. This mutant replaces exon 1 of Dop1R1 with a Gal4; however, an in-frame start codon exists in exon 2, and this truncated product has been shown to be functional in cell culture (*79*). A different Dop1R1 mutant (not assayed in this study) generated using a similar technique (replacement of exon 1) has been shown to have detectable protein product in the brain via immunohistochemistry (*43*). The two other Dop1R1 mutants we tested, which have been verified to be either null (*43*) or a strong hypomorph (*48*, *49*), are included in Fig. 8A and have strong effects on egg-laying choice.

### High-throughput egg-laying choice chamber

Experiments with wild-type flies or mutants were conducted in the same manner as described in a previous study (*16*). Experiments with Kir2.1 flies were conducted in the same manner to Kir2.1* assays from a previous study (*16*), except flies were pretreated at 18°, 27°, 29°/29.5°, or 31°C instead of only at 18° or 31°C. Experiments with *D. suzukii* flies were with 1.2% agarose instead of 1% agarose because pilot experiments suggested that this species laid more eggs on agarose with a higher percentage (i.e., harder agarose). DL, TUT, and *D. suzukii* females were mated with males of the same strain/species. Dimensions and design of all chambers used in this study are shown in fig. S1.

### Gluing sensory organs

A thin layer of blue light–cured glue (Bondic) was applied to sensory organs under gentle cold anesthesia. For the tarsi, only the distal two segments were lightly covered with glue. For the proboscis, the rostrum, proboscis, and labellum were glued such that the proboscis could not extend at all. Egg-laying assays were started 3 to 5 hours after glue application.

### Latency to feeding assay

Flies were treated as in egg-laying assays except flies were mated in a bottle with only plain 0.8% agarose (a source of water, but not food). This treatment deprived flies of food for 26 hours, allowing initiation of feeding to be measured as a proxy for the ability to sense sucrose. Groups of three to six females were placed on the 0 mM side of a 0 versus 200 mM chamber under gentle cold anesthesia. These chambers were like the standard two-choice egg-laying chambers, except a sliding plastic barrier prevented flies from entering the 200 mM side of the chamber (fig. S1H), and both ethanol and acetic acid were omitted such that sucrose was the only food source. Flies were imaged from above at 15 frames per second with a CM3-U3-13Y3M Chameleon camera (FLIR) (Fig. 7B) or from below at 15 frames per second using an ORCA-Fusion C14440-20UP camera (Hamamatsu) (Fig. 8B). 850-nm light-emitting diodes (LEDs) illuminated the arena from the opposite side of the camera. Both configurations could detect proboscis extension or feeding posture, and the configuration remained consistent within a given figure panel. A few minutes after starting image acquisition, we slid out the plastic barrier to allow flies to freely cross over to the 200 mM side of the chamber. The latency from the first time a tarsus crossed over the barrier to proboscis extension or entrance into a feeding posture (fly is still with head tilted down and forelegs forward) (*80*) was scored manually (fig. S6). All flies that were scored as entering a feeding posture were also extending their proboscis once the proboscis became unobstructed by the head. Flies rarely, if ever, extended their proboscis or entered a feeding posture on 0 mM before crossing to 200 mM or when crossing from 0 to 0 mM in control chambers.

### Immunostaining and microscopy

The brain and ventral nerve cord of 2- to 5-day-old females were dissected and processed similarly to a previous study (*81*). Images were taken with an LSM 780 confocal microscope (Zeiss).

### Automated determination of egg-laying search

The egg-laying search was analyzed as described in a previous study (*16*). We used an analysis of locomotor speed to define the start of the search (see below) and egg deposition to approximate the end of the search [which in free-walking flies in these chambers occurs only a few seconds after the search is concluded and the final abdomen bend to lay an egg is initiated (*16*)]. The start of the search period was determined for each egg by smoothing the locomotor speed trace before egg deposition with an 18.5-s boxcar filter and identifying the first frame before an egg deposition event where this smoothed signal dropped below 0.1 mm/s. The minimum search duration was thus 9 s because of the length of the boxcar filter. These parameters were determined empirically to yield search onset times consistent with visual inspection of the data.

### Calculation of egg-laying rate as a function of time

Egg-laying rate functions were calculated in the same manner as described previously (*16*). Data from all flies tested in a given chamber type were combined before any calculations. First, we iterated through each time bin denoted on the *x* axis and, for each bin, we counted the number of egg deposition events that were assigned to that bin, #_eggs_(bin). Second, we iterated through the same time bins and counted the number of video frames in which flies were assigned to that bin, #_frames_(bin), during an egg-laying search period. Third, we iterated through the same time bins and counted the number of times flies changed assignment into that bin, #_visits_(bin), during an egg-laying search period (i.e., we did not keep incrementing the “visits” counter if the fly remained in a time bin from one frame to the next).

To get the mean egg-laying rate, we calculated #_eggs_/#_frames_ for each bin. Because videos were recorded at 2 frames per second, we multiplied the value for each bin by 120 to convert to units of eggs per minute.

To get the confidence interval per bin, we used the Clopper-Pearson method (“exact” binomial confidence interval) to calculate the 90% confidence interval for #_eggs_/#_visits_ for each bin. We then converted the confidence interval for each bin to units of eggs per minute by multiplying by 120*#_visits_/#_frames_ per bin. The confidence interval cannot be directly calculated from #_eggs_/#_frames_ because then the confidence interval would be dependent on the video frame rate. Confidence interval and *P* value calculations (see the “Statistics” section) for egg-laying rate functions assume that each individual search and each substrate transition within a search are independent. We argue that individual searches can be reasonably considered independent because flies prepare (ovulate) each egg individually before searching for an egg-laying site and egg-laying events are separated in time by at least 1 min (*16*). Within an individual search event, substrate transitions can be approximated as independent because a fly would have to experience a different substrate before remaking a specific substrate transition. We introduce these nonideal assumptions because we needed to combine data across many searches and flies to quantitatively analyze the impact of substrate history on egg laying across multiple dimensions (e.g., different substrates and time). All our stated conclusions are conservative in that we only focus on statistically significant results if egg-laying rate curves are also obviously different by eye.

For these rate functions, search periods with duration of less than 30 s were set to 30 s. This prevented very short search periods from introducing fluctuations in the rate curves (by contributing to the numerator and not contributing much to the denominator). Hence, rate curves varied less from replicate to replicate or condition to condition. Note that search periods already had a minimum duration of 9 s as automatically generated by the search period calculation (Materials and Methods). Analyzing the data with no minimum search duration (or with other definitions of the search period) did not change any of the stated conclusions (fig. S2). Binning the *x* axis in different ways also did not qualitatively change any of our stated conclusions.

Here, we examine egg-laying rates as a function of time since visiting another substrate as our main approach for extracting algorithmic insight. As with any simple analysis, some important variables may be ignored (e.g., the locomotor vigor of the search bout, the duration of the visit to the previously visited substrate, and visits to other substrates). These variables can vary in different chamber types, and hence, there are fluctuations in our egg-laying rate plots whose origin is not yet known. Nonetheless, this analysis allowed us to intuitively capture how the history of substrate experiences affects the current egg-laying behavior.

### Mathematical models that attempt to transform sensory input to egg-laying rate

We tested two models to better understand how sucrose sensory information might be transformed into egg-laying rates. Analysis of these models highlights that using pure sucrose sensory information (i.e., a simple time history of sucrose concentration rather than a computation involving an internal expectation) fails to explain our experimental data.

In the first model (model #1, fig. S4, A and B), past sucrose concentration history is convolved with a kernel to generate the current egg-laying rate. A kernel that weighs previous sucrose history positively (with a long tail) and more recent sucrose history negatively can roughly approximate the average egg-laying rate curves in a 0 mM versus 200 mM chamber (kernel not shown graphically). We calculated kernels using code and concepts from (*82*). The kernel just described essentially represents an “average_historical_[sucrose] − current_[sucrose]” calculation and resembles kernels for bacterial chemotaxis (*58*, *83*), but over a longer time scale. If the average [sucrose] in recent history is high (i.e., the fly has spent a lot of time on 200 mM) and the current [sucrose] is low (i.e., the fly is currently on 0 mM), then the egg-laying rate would be high. The area under the kernel is 0, allowing for perfect adaptation (i.e., reaching a set point egg-laying rate once the fly has spent enough time on any substrate). However, the kernel generated to match the average egg-laying rates in a 0 mM versus 200 mM chamber qualitatively fails in a 0 mM versus 500 mM chamber (e.g., it overestimates the average egg-laying rate upon transition from 500 to 0 mM). This failure can be attributed to the fact that the kernel is convolved with [sucrose] rather than using a comparison of [sucrose] with an internal expectation of [sucrose]. With this modification—e.g., replacing [sucrose] with “is([sucrose] ≥ [best_sucrose])”—the model can better approximate the very similar average egg-laying rate curves in 0 mM versus 200 mM, 0 mM versus 500 mM, and 200 mM versus 500 mM chambers (Fig. 2A) (*16*). However, even after this modification, the model has significant failures. For example, the drop in egg-laying rate upon transitioning onto the low relative value option is slow, but in experimental data, it is immediate (Fig. 2A) (*16*).

We also assessed a second model that is capable of implementing more rapid changes in the egg-laying rate following substrate transitions. In this model, the fly’s egg-laying rate is adjusted on the basis of a comparison of the current egg-laying rate to a set point, or desired, egg-laying rate (model #2, fig. S4, C and D) and the current [sucrose]. Conceptually, this model has features of an integral controller (*84*) that brings the egg-laying rate back to a set point after an environmental sucrose perturbation. This model fails to capture the fact that egg-laying rate curves are similar on the lower relative option in two-choice chambers (Fig. 2A) (*16*), i.e., they are not related to the absolute difference in sucrose concentration of the options, as is the case in this model. Just like model #1, if one replaced [sucrose] with “is([sucrose] ≥ [best_sucrose]),” this model would do better. While this model can more rapidly decrease egg-laying rates upon entry to the lower relative value option, it would still have important limitations, even after the modification. For example, the model would require an assumption that flies have an egg-laying rate drive during a search, an extra computation that we do not have neurophysiological evidence for as of yet. Moreover, even with our proposed modification, neither model would be able to explain why flies have a higher egg-laying rate on the second best option (compared to the third best) in scenarios where they make faster decisions (Fig. 4D). In other words, we are not quite sure whether flies explicitly remember only the best option and make perfect comparisons to it––which would not predict that experimental result––or whether the expectation or comparison takes another form (see Discussion for forms the expectation might take). Rather than pursue these hypothetical models in depth here, we aim to generate neural signal–driven models down the road, once more is known at the neural level, beyond the oviDN rise-to-threshold signal (*16*).

### Statistics

We used the two-sided Wilcoxon rank sum test to calculate all *P* values. *P* values for egg-laying rate curves and first *P* value in the main text are comparing the number of trials with (or without) events in two separate groups. For a single group, trials with an event are treated as 1, and trials without an event are treated as 0. Then, the two groups (each a set of 0 and 1) are compared using the two-sided Wilcoxon rank sum test (*P* values calculated using the two-sided Fisher’s exact test are similar and similarly significant, except the second *P* value in Fig. 3C is 0.058). Note that these *P* value calculations assume that trials are independent (see the “Calculation of egg-laying rate as a function of time” section for discussion of assumptions). The second and third *P* values in the main text compare the distribution of pause times—the time difference between egg deposition and when the 1-s boxcar smoothed locomotor speed increases above 0.1 mm/s before egg deposition. *P* values in Fig. 3D are calculated by comparing the distributions of dwell times on sucrose before egg laying on plain. The latter two *P* values assume independence of pause times for egg laying and dwell times before egg laying, which are both reasonable because eggs are laid individually. All other *P* values are comparing two groups of individual flies, rather than individual trials.

Error bars are SEM unless otherwise described. For egg-laying choice fractions (like Fig. 1D or fig. S3A), means are fraction of eggs laid on the lower sucrose option after all eggs from all flies are pooled and error bars indicate the 95% confidence interval of this fraction calculated using the Clopper-Pearson method (exact binomial confidence interval), which assumes that all egg-laying events are independent. A dotted line is drawn at 0.5 for all egg-laying preference and positional preference plots. Note that if flies were to have no preference for laying eggs on one substrate over the other, their egg fraction might fall off the 0.5 line because of a positional preference to spend more time on one substrate over the other, usually sucrose. For all experiments, no data were excluded––except one Dop1R1 mutant line that may still have functional Dop1R1 protein product (see “Flies”)––, and no statistical method was used to choose sample size.

### Data analysis software

All data analyses were done using MATLAB (MathWorks).

## Notes

View/request a protocol for this paper from *Bio-protocol*.
