# Supplementary material for: An internal expectation guides Drosophila egg-laying decisions
Source: Sci Adv. 2022 Oct 28;8(43):eabn3852. doi: 10.1126/sciadv.abn3852 (PMC9616500; doi:10.1126/sciadv.abn3852)
Supplement: Supplementary file 1 — Figs. S1 to S7 [file sciadv.abn3852_sm.pdf]

Science Advances
AAAS

Supplementary Materials for

An internal expectation guides *Drosophila* egg-laying decisions

Vikram Vijayan *et al.*

Corresponding author: Vikram Vijayan, vvijayan@rockefeller.edu; Gaby Maimon, maimon@rockefeller.edu

Sci. Adv. **8**, eabn3852 (2022)
DOI: 10.1126/sciadv.abn3852

The PDF file includes:

Figs. S1 to S7
Legends for tables S1 and S2
Legends for movies S1 to S3

Other Supplementary Material for this manuscript includes the following:

Tables S1 and S2
Movies S1 to S3

Science Advances
AAAS

Supplementary Materials for

An internal expectation guides *Drosophila* egg-laying decisions

Vikram Vijayan*, Zikun Wang, Vikram Chandra, Arun Chakravorty, Rufei Li, Stephanie L. Sarbanes, Hessameddin Akhlaghpour, and Gaby Maimon*

*Corresponding author. Email: vvijayan@rockefeller.edu, maimon@rockefeller.edu

This PDF file includes:

Figs. S1 to S7
Legend for Tables S1 to S2
Legend for Movies S1 to S3

Other Supplementary Materials for this manuscript include the following:

Tables S1 to S2
Movies S1 to S3

Table S1. Summary of all tracking data.

Table S2. Summary of screen for egg-laying choice related neurons. All data in Fig. 7A is reported in increasing order of the x-axis in Fig. 7A. Data for 6 additional control lines that are not in Fig. 7A are also reported and marked ‘control’.

Movie S1. Example egg laying event in a 0/500/200/500/200 mM sucrose (bottom to top) chamber at 10X speed.

Movie S2. Example egg laying event in a 0/200 mM sucrose (bottom to top) 2 mm tunnel w/ bounce chamber at 10X speed.

Movie S3. Example egg laying event in a 500/0/500 mM sucrose (bottom to top) chamber at 10X speed.

Fig S1.

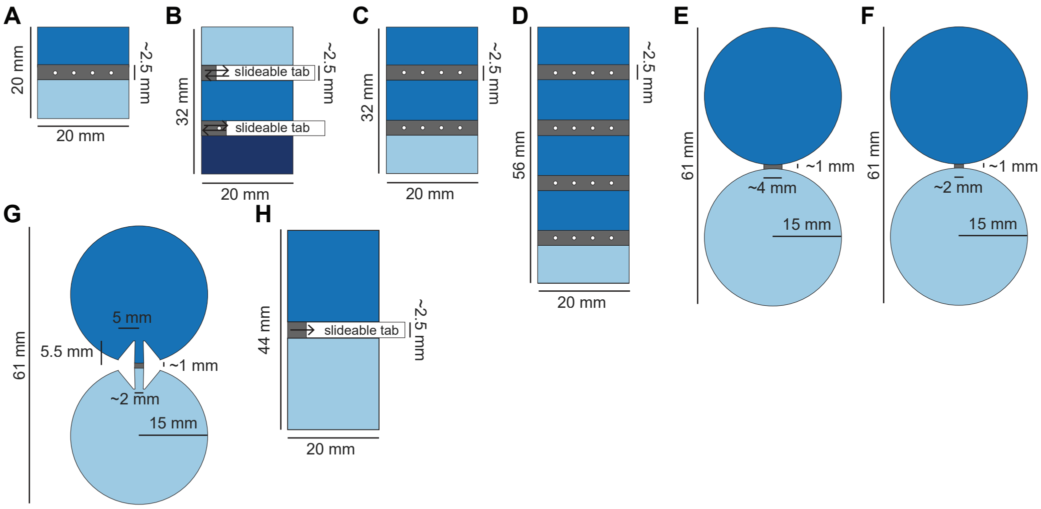

Fig. S1. Egg-laying choice chambers used in this study. (**A**) Two-island chamber. (**B**) Three-island chamber with movable boundaries used in Fig. 1D-E. (**C**) Three-island chamber. (**D**) Five-island chamber. (**E-G**) restricted corridor (and control) chambers used in Fig. 3A-F. (**H**) Movable boundary chamber used in Fig. 7B and Fig. 8B.

Fig S2.

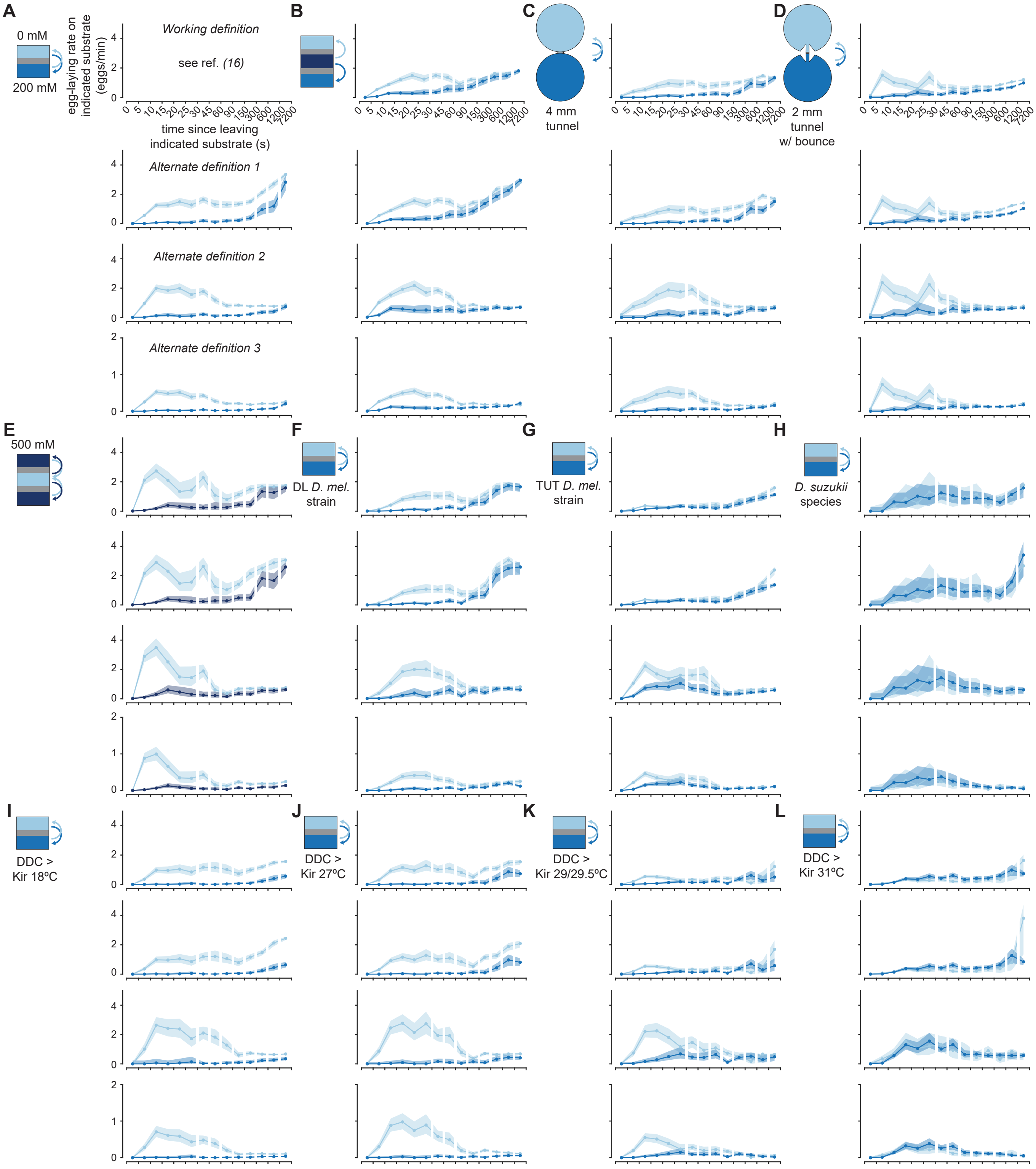

Fig. S2. Egg-laying rate functions with different definitions of the *search* period. (**A-L**) Egg-laying rate functions in twelve chambers with different search-period definitions. Egg-laying rate curves in panel A use data from ref. (*16*). *Working definition*: Egg-laying rate functions as defined in this study and a previous study (*16*) (Methods). *Alternate definition 1*: Egg-laying rate functions with search durations under 30 s not set to 30 s. *Alternate definition 2*: Egg-laying rate functions with the search period always set to the 90 s before each egg-laying event. If this 90 s extends into the previous egg-deposition event plus 1 minute, it was shortened. This prevents search periods from overlapping with other search periods or with ovulation (which takes at least 1 minute) (*16*). *Alternate definition 3*: Same as *alternate definition 2* except we changed 90 s to 15 min.

Fig S3.

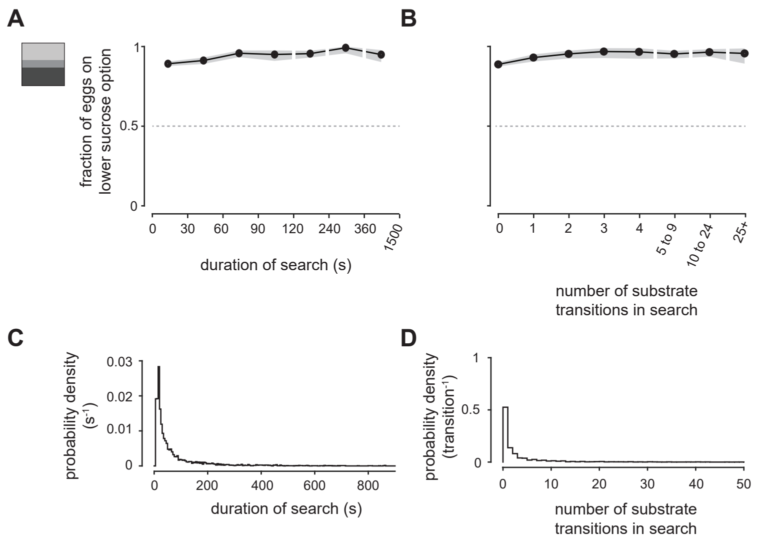

Fig. S3. Egg-laying behavior as a function of search duration. (**A**) Fraction of eggs on the lower sucrose option as a function of search duration with 95% confidence interval. (**B**) Fraction of eggs on the lower sucrose option as a function of number of transitions during search with 95% confidence interval. (**C**) Distribution of search durations. (**D**) Distribution of number of transitions per search. In panels A-D, data was combined from 0/200, 0/500, and 200/500 mM chambers. 3979 eggs from 95 flies.

Fig S4.

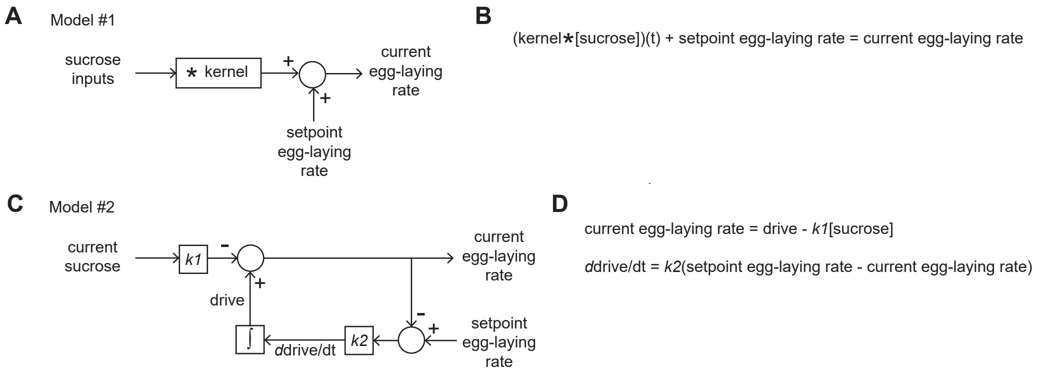

Fig. S4. Two models without a comparison to an internal expectation that fail to reproduce experimental data. (**A**) Model #1 is a model where sucrose inputs are convolved with a kernel to generate the current egg-laying rate. (**B**) Explicit mathematical form of model diagrammed in panel A. (**C**) Model #2 is a model where the current sucrose input as well as a comparison of the current egg-laying rate with a setpoint control the current egg-laying rate. (**D**) Explicit mathematical form of model diagrammed in panel C. See Methods for detailed discussion of both models including why they fail and how these models can better approximate experimental data if [sucrose] is replaced with a comparison to an internal expectation.

Fig S5.

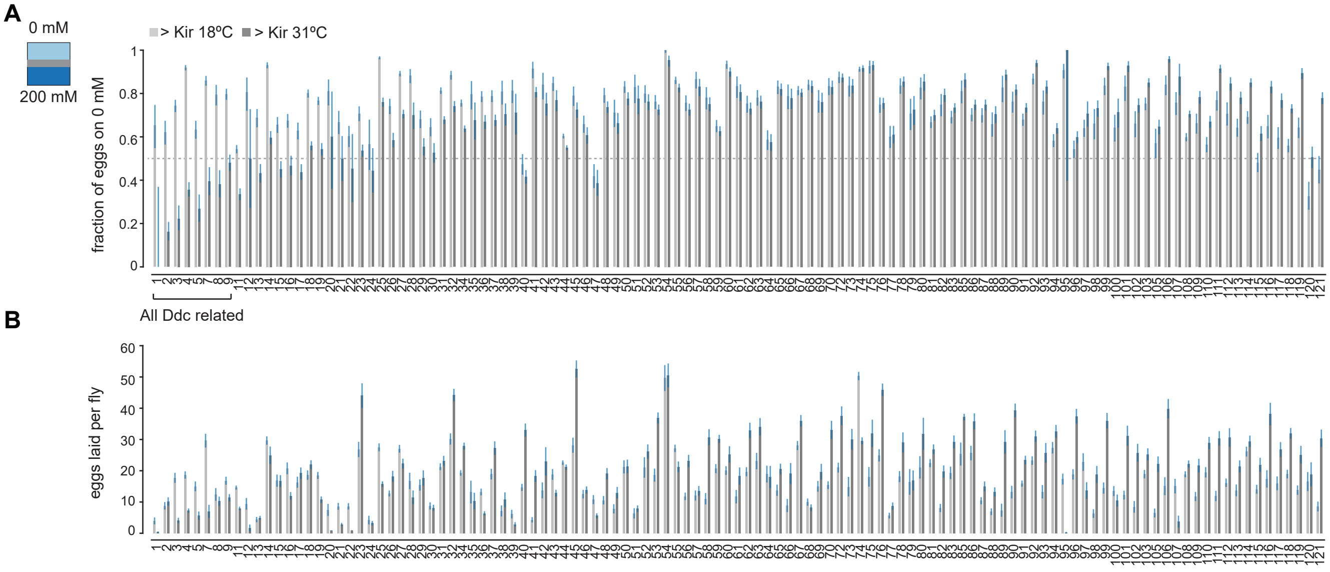

Fig. S5. Summary of screen. (**A**) Fraction of eggs on 0 mM with 95% confidence interval. Data is in increasing order of the x-axis in Fig. 7A. Genotype names are indicated in Table S2. **(B**) Eggs laid per fly as in panel A.

Fig S6.

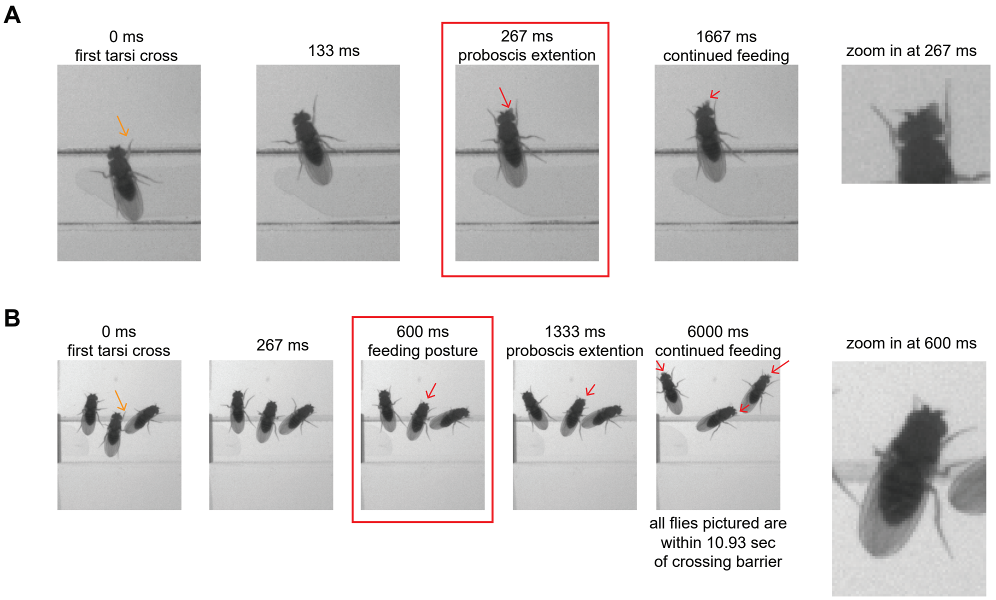

Fig. S6. Example annotation of feeding events. (**A**) Example annotation of a DDC > Kir (31°C pre) fly crossing the barrier from plain to 200 mM sucrose (orange arrow) and initiating proboscis extension (red square). The difference in time between the red square and orange arrow is the time used in Fig. 7B. (**B**) Example annotation of a DDC > Kir (31°C pre) fly crossing the barrier from plain to 200 mM sucrose (orange arrow) and entering a feeding posture (red square). The difference in time between the red square and orange arrow is the time used in Fig. 7B. Proboscis extension events are invariably observed in subsequent frames once the proboscis is unobstructed by the head.

Fig S7.

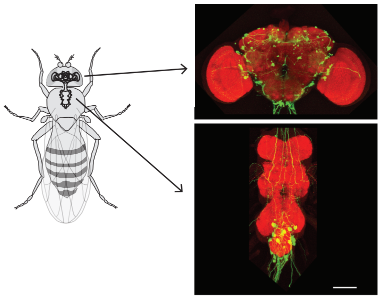

Fig. S7. Neurons labeled by DDC-Gal4 in a female fly. Amplified EGFP signal (green) and nc82 counterstain (red). Fly genotype is w-; UAS 2xEGFP/+; DDC-Gal4/+. Scale bar, 100 µm.
